# Efficacy and safety of activated prothrombin complex concentrate for the reversal of vitamin K antagonist major bleeding

**DOI:** 10.1038/s41598-022-05803-w

**Published:** 2022-02-02

**Authors:** Marwan Sheikh-Taha, R. Monroe Crawley

**Affiliations:** 1 Department of Pharmacy Practice, Lebanese American University, Byblos, Lebanon; 2 Department of Pharmacy, Huntsville Hospital, Huntsville, AL USA

**Keywords:** Cardiology, Medical research

## Abstract

Data on the use of activated prothrombin complex concentrate (aPCC) for the management of warfarin associated major bleeding is sparse. The objective of the study was to assess the achievement of effective clinical hemostasis using aPCC in patients presenting with major bleeding while on warfarin. We also assessed the safety of the drug. This retrospective study was conducted at a tertiary care teaching center in the USA where patients with major bleeding while receiving warfarin, and received aPCC were included. Efficacy of aPCC in achieving effective hemostasis was assessed according to the International Society of Thrombosis and Hemostasis Scientific and Standardization Subcommittee criteria. Efficacy was also assessed by achieving INR < 1.5 after treatment. The primary safety endpoint was the occurrence of any thromboembolic complications. A total of 67 patients were included in the study. The most common site for bleeding was intracerebral hemorrhage (n = 37, 55.2%), followed by gastrointestinal bleed (n = 26, 38.8%). Clinical hemostasis was achieved in 46 (68.7%) patients and of the 21 (31.3%) patients who did not achieve clinical hemostasis, 16 died. Thirty nine (58.2%) patients achieved INR < 1.5. Five (7.5%) patients developed thromboembolic complications. This study suggests that the use of aPCCs is effective in achieving effective hemostasis in patients on warfarin presenting with major bleeding.

## Introduction

The vitamin K antagonist (VKA), warfarin, is used in a variety of clinical settings including the treatment, prevention and reduction of recurrent venous thromboembolism, and stroke prevention in patients with atrial fibrillation (AF)^1^. As with other anticoagulants, the use of warfarin is associated with an increased risk of bleeding and serious or life-threatening bleeding requiring rapid, full reversal of any warfarin effect.

Phytonadione (vitamin K) in combination with prothrombin complex concentrate (PCC) administration is recommended by the American College of Chest Physicians for the reversal of VKAs with 4-factor PCC (4PCC) recommended as first-line therapy over fresh frozen plasma (FFP)^2^. 4PCC contains inactive factors II, VII, IX, and X. Additionally, 4PCC contains small amounts of heparin and protein C and S^3^.

Activated PCC (aPCC) contains inactive factors II, IX, and X, but in contrast to 4PCC, contains activated factor VII, and FEIBA (Baxter, Westlake, CA) is the only commercially aPCC in the USA. aPCC is comparable to previously studied combination regimens consisting of 3-factor PCC (3PCC) and recombinant factor VIIa (rFVIIa)^3^.

Studies comparing 3PCC + rFVIIa with 4PCC found that 3PCC + rFVIIa achieved greater reductions in International Normalization Ratio (INR) compared to 4PCC, although both regimens were able to achieve an INR < 1.5. Additionally, these studies found that 3PCC + FVIIa had tenfold higher incidence of deep venous thrombosis (DVT) compared to 4PCC^4^. Extrapolations from these findings, along with previous hemophilia-related data, have led to concerns that aPCC therapy carries inherently increased risks for coagulopathies. However, lower doses of aPCC have been evaluated for the treatment of emergent, VKA-related bleeding. Researchers found no difference in the ability or time to achieve a post-treatment INR < 1.5. Furthermore, they found no difference in post-treatment thrombotic complications when comparing aPCC and 4PCC^5^.

Since data on the use of aPCC for VKA associated major bleeding is sparse, we conducted this study to assess the efficacy and safety of aPCC in patients presenting with major bleeding while on the VKA, warfarin.

## Methods

This is a single center, retrospective analysis conducted between January 2019 and December 2020 at a tertiary care teaching center, Huntsville Hospital, Alabama, USA. Patients were eligible for inclusion if they were ≥ 18 years of age and presented with major bleeding while receiving warfarin, and received the aPCC, FEIBA (Anti-Inhibitor Coagulant Complex, Takeda). Patients with INR elevations contributable to other causes, such as chronic cirrhosis, acute hepatotoxicity, or shock liver, were excluded. As per hospital protocol, FEIBA was given intravenously at a dose of 500 units if the INR was < 5, and 1000 units if INR ≥ 5. In addition, all patients received intravenous vitamin K at a dose of 10 mg (Supplementary Information).

Major bleeding was defined according to International Society of Thrombosis and Hemostasis (ISTH) definition for major bleeding in nonsurgical patients as (1) fatal bleeding, (2) symptomatic bleeding in a critical area or organ, (3) bleeding causing a fall in hemoglobin level of ≥ 2 g/dL, or leading to transfusion of ≥ 2 units of whole blood or red cells^6^. The efficacy of aPCC in achieving effective hemostasis was assessed according to ISTH Scientific and Standardization Subcommittee criteria^7^. The efficacy was also assessed by achieving INR < 1.5 after administration of aPCC. Baseline INR was obtained for each patient prior to aPCC administration with follow-up INRs at 30 min and one hour after administration. The primary safety endpoint was the occurrence of any thromboembolic complication after treatment with aPCC including myocardial infarction, stroke, transient ischemic attack, DVT, and pulmonary embolism (PE).

Data were collected from the medical records of patients, including demographic information, physicians’ orders and notes, laboratory values, and any other relevant details. Patients were followed until discharge or death. Waiver of informed consent and ethical approval for this study was obtained from Huntsville Hospital’s Institutional Review Committee and all methods were performed in accordance with the relevant guidelines and regulations. Data were analyzed with descriptive statistics and frequency distributions.

Statistical analyses were conducted with SAS (version 9.4, SAS Institute Inc, Cary, NC). Normality was assessed for continuous variables by the Shapiro–Wilk test, and the Kolmogorov–Smirnov test. All continuous data was found to be non-Gaussian; thus, the Mann Whitney U test was used to assess statistical differences between groups. All categorical variables were evaluated using Chi Squared and Fisher’s Exact as appropriate.

### Ethics approval and consent to participate

Ethical approval for this study was obtained from Huntsville Hospital’s Institutional Review Committee.

## Results

A total of 67 patients met the inclusion criteria during the study period and were included, of which 41 were males (61.1%) and 26 (38.9%) were females. The mean age ± SD was 72.8 ± 10.6 years. The most common indication for warfarin use was AF (n = 32, 47.8%), followed by mechanical valve (n = 15, 22.4%), and DVT/PE (n = 12, 17.9%). The most common site for bleeding was intracerebral hemorrhage (ICH) (n = 37, 55.2%), followed by gastrointestinal (GI) bleed (n = 26, 38.8%). Table 1 describes patient demographic and clinical characteristics. Nineteen patients had an INR ≥ 5 upon admission and received aPCC at a dose of 1000 units while the remaining 48 patients had INR values < 5 and received 500 units. Effective hemostasis was achieved in 46 (68.7%) patients and of the 21 (31.3%) patients who did not achieve hemostasis, 16 died. Thirty nine (58.2%) patients achieved an INR < 1.5 post treatment with aPCC, and four patients died before a repeat INR was obtained. Table 2 describes bleeding management outcomes, Table 3 describes INR values upon admission and after receiving aPCC, and Table 4 describes characteristics of patients who died.

**Table 1:** Patient characteristics.

	n	%
**Age (years)**		
Mean ± SD	72.8 ± 10.6	
**Sex**		
Male	41	61.1
Female	26	38.9
**Indication for warfarin**		
AF	32	47.8
Mechanical valve	15	22.4
DVT/PE	12	17.9
Ischemic stroke	5	7.5
Peripheral arterial disease	2	2.9
APS	1	1.5
**Bleeding site**		
ICH	37	55.2
GI	26	38.8
Retroperitoneal	3	4.5
Aortic dissection	1	1.5
**Length of hospital stay (days)**		
Mean (range)	8.6 (1–40)	

*APS *antiphospholipid syndrome*, DVT* deep venous thrombosis, *GI *gastrointestinal*, ICH* intracerebral hemorrhage*, PE* pulmonary embolism.

**Table 2:** Bleeding management outcome.

Bleeding location	Clinical hemostasis	
	Yes n (%)	No n (%)
ICH	26 (70.3)	11 (29.7)
GI	19 (73.1)	7 (26.9)
Retroperitoneal	0 (0)	3 (100)
Aortic dissection	1 (100)	0 (0)
Total	46 (68.7%)	21 (31.3%)

*ICH* intracerebral hemorrhage, *GI* gastrointestinal.

**Table 3:** Mean INR values before and after receiving aPCC.

Baseline INR Mean ± SD/(range)	INR after 30 min Mean ± SD/(range)	INR after 60 min Mean ± SD/(range)	Goal INR achieved
4.6 ± 3.7 (1.7–13.6)	1.7 ± 0.6 (1.1–4.4)	1.48 ± 0.5 (1.1–4.0)	39 (58.2%)

**Table 4:** Details of deaths during hospitalization.

Patient	Age	Sex	Indication for warfarin	Bleeding site	Target INR achieved	Other hemostatic agents given	Day of death after aPCC
1	69	M	AF	GI	Yes		3
2	74	M	Mechanical valve	ICH	Yes	FFP, platelets	3
3	70	F	DVT/PE	ICH	Yes		13
4	56	M	AF	Retroperitoneal	No	Additional dose of aPCC	2
5	58	F	APS	ICH	Yes		2
6	63	M	AF	Retroperitoneal	No	FFP, platelets	1
7	78	M	AF	ICH	Yes		2
8	82	M	Mechanical valve	GI	No		1
9	84	M	AF	ICH	Unknown*		1
10	80	M	AF	ICH	Yes	FFP	10
11	69	F	AF	GI	Unknown*		1
12	77	M	AF	ICH	Unknown*		1
13	70	F	AF	ICH	Unknown*	FFP	1
14	75	F	AF	GI	No	FFP	17
15	87	M	AF	ICH	Yes		8
16	74	F	AF	ICH	Yes		3

*AF* atrial fibrillation, *APS* antiphospholipid syndrome, *DVT* deep venous thrombosis, *FFP* fresh frozen plasma, *GI* gastrointestinal, *ICH* intracerebral hemorrhage, *PE* pulmonary embolism.

*Patient died before repeat INR.

Of the 67 patients meeting inclusion criteria, 21 (31.3%) patients had total body weights (TBW) greater than 120% of their ideal body weight (IBW). These patients were classified as obese. In these patients, 14 (66.7%) achieved hemostasis, 14 (66.7%) achieved INRs < 1.5, 2 (9.5%) experienced an ADR, and 6 (28.6%) died. When comparing these patients to those with TBW less than 120% of their IBW, obese patients had similar average percentage change in INR than non-obese patients (− 0.49 vs − 0.58, p = 0.11). There were no differences seen when comparing obese patients to non-obese patients in achievement of hemostasis (64.5% vs 72.2%, p = 0.498), ability to achieve INR < 1.5 (55.6% vs. 50%, p = 0.6622), or mortality (29% vs 27.8%, p = 0.91).

In addition to aPCC, patients received additional management to control bleeding; 15 patients received FFP, and seven patients who were on anti-platelet therapy or had thrombocytopenia received platelet transfusions. After receiving aPCC, five patients (7.5%) developed thromboembolic complications (three of them received FFP in addition to aPCC); four patients developed DVT (one on day four of admission, two on day five, and one on day 20), and one patient developed PE on day 15 of admission.

## Discussion

The use of aPCC for the reversal of warfarin has not been firmly confirmed in large scale randomized controlled trials. This study assessed the achievement of effective hemostasis using aPCC in patients on chronic warfarin therapy presenting with major bleeding. While few available studies defined efficacy as achieving posttreatment INR <1.5, in this study other than assessing posttreatment INR, we assessed achieving effective hemostasis, a clinical endpoint, according to ISTH Scientific and Standardization Subcommittee criteria.

In this study, 68.7% of patients achieved hemostasis after receiving aPCC which is comparable to the results reported by Wojciket et al., where the rates of successful hemostasis (defined as patients who survived the bleeding episode) was 77.8% when aPCC was used at a dosing regimen similar to the one used in this study, and 62.5% as reported by Rowe et al.^5,8^.

Achievement of INR < 1.5 was achieved in 58.2% of patients. This is similar to results reported by Rowe et al., where the percentage of patients achieving INR < 1.5 was 57.9%^5^. Incongruence between hemostasis and achievement of an INR goal is to be expected. Hepatic insufficiency, increased phytonadione consumption, and drug interactions can alter INR results in patients with life-threatening bleeding. The goal of anticoagulation reversal with VKAs should include goals balanced between hemostasis and a specific INR goal, rather than with either outcome alone.

Due to the high levels of coagulation factors contained in aPCC, the thrombotic complications associated with its use remain a concern. In this study, five patients (7.5%) developed thrombotic complications, three of whom received FFP along with aPCC, similar to the findings of a study involving 388 patients where 4PCC was used (7.3%)^9^. On the other hand, in a meta-analysis of 1032 patients where 4PCC was used to reverse the effect of VKA, the incidence of thromboembolic events was 1.8%^10^. The addition of FFP to aPCC could have significantly increased the risk of thromboembolic events in this study.

When analyzing obese patients, we found results similar to those in recent studies. McKinney, et al. reported significantly higher proportions of non-obese patients achieving INR values of 1.4 or less. However, these lower INR reversal rates were not associated with lower rates of hemostasis^11^.

Our study has several limitations. This was a single center retrospective chart review with a small sample and without a comparator group. Moreover, some patients received FFP and platelet transfusions which can influence the outcomes. In addition, the dose of aPCC used in the study is based on limited data and may not be the optimal dosing regimen. Furthermore, we were not able to follow patients after discharge from the hospital which may underrate the number of thrombotic events. Nevertheless, despite these limitations, this study adds novel and important information involving the use of aPCC for the reversal of warfarin.

## Conclusion

This study suggests that the use of aPCC is effective in achieving effective hemostasis in patients on warfarin presenting with major bleeding and thrombotic complications associated with its use remain a concern. Further controlled studies are needed to confirm these findings and conclude the optimal dosing regimen for maximal efficacy and safety.

## Supplementary Information

Supplementary Information 1.

## Abbreviations

AF: Atrial fibrillation
aPCC: Activated PCC
DVT: Deep venous thrombosis
FFP: Fresh frozen plasma
GI: Gastrointestinal
ISTH: International Society of Thrombosis and Hemostasis
PE: Pulmonary embolism
PCC: Prothrombin complex concentrate
rFVIIa: Recombinant factor VIIa
VKA: Vitamin K antagonist
