# Supplementary figures and images for: Efficacy and safety of activated prothrombin complex concentrate for the reversal of vitamin K antagonist major bleeding
Source: Sci Rep. 2022 Feb 2;12:1814. doi: 10.1038/s41598-022-05803-w (PMC8810750; doi:10.1038/s41598-022-05803-w)

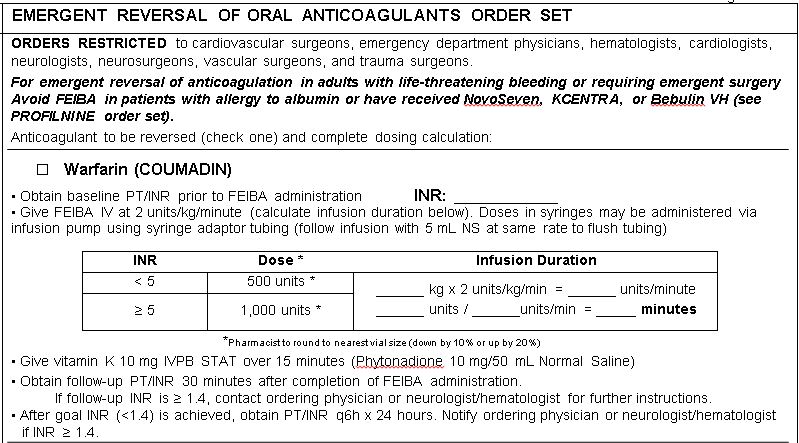

Supplement: Supplementary file 1 — Supplementary Information 1. [file 41598_2022_5803_MOESM1_ESM.jpg]
